# Injectable Thermosensitive Chitosan Solution with *β*-Glycerophosphate as an Optimal Submucosal Fluid Cushion for Endoscopic Submucosal Dissection

**DOI:** 10.3390/polym13111696

**Published:** 2021-05-22

**Authors:** Seung Jeong, Han Jo Jeon, Kyoung-Je Jang, Sangbae Park, Hyuk Soon Choi, Jong Hoon Chung

**Affiliations:** 1 Department of Biosystems & Biomaterials Science and Engineering, Seoul National University, Seoul 08826, Korea; jsw3055@snu.ac.kr (S.J.); sb92park@snu.ac.kr (S.P.); 2 Division of Gastroenterology and Hepatology, Department of Internal Medicine, Korea University College of Medicine, Seoul 02841, Korea; roadstar82@naver.com; 3 Division of Agro-system Engineering, College of Agriculture and Life Science, Gyeongsang National University, Jinju 52828, Korea; kj_jang@gnu.ac.kr; 4 Institute of Agriculture & Life Science, Gyeongsang National University, Jinju 52828, Korea; 5 Department of Biosystems Engineering, Seoul National University, Seoul 08826, Korea; 6 Research Institute of Agriculture and Life Sciences, Seoul National University, Seoul 08826, Korea; 7 BK21 Global Smart Farm Educational Research Center, Seoul National University, Seoul 08826, Korea

**Keywords:** thermosensitive chitosan solution, *β*-glycerophosphate, endoscopic submucosal dissection (ESD), submucosal fluid cushion, submucosal injection agent

## Abstract

Endoscopic submucosal dissection (ESD) is a surgical procedure to remove early neoplastic lesions in the gastrointestinal tract with the critical issue of perforation. A submucosal fluid cushion, such as normal saline, is used as a cushioning agent to prevent perforation; however, its cushioning maintenance is insufficient for surgery. In this study, we introduce an injectable thermosensitive chitosan solution (CS) with *β*-glycerophosphate (*β*-GP) as a submucosal injection agent for ESD. The CS/*β*-GP system with optimal *β*-GP concentration showed drastic viscosity change near body temperature while other commercial products did not. Additionally, the injectability of the solution was similar to or greater than other commercial products. The solution with low *β*-GP concentration showed low cytotoxicity similar to other products. An in vivo preclinical study illustrated maintenance of the high cushioning of the thermosensitive solutions. These results indicate that a CS/*β*-GP system with optimal *β*-GP concentration might be used as a submucosal injection agent in ESD, and further studies are needed to validate the effectiveness of the solutions in vivo.

## 1. Introduction

Endoscopic submucosal dissection (ESD) is a minimally invasive endoscopic procedure that removes an early lesion of the gastrointestinal (GI) tract using electrocautery [1,2]. However, complications of bleeding (~15.6%) and perforation (~9.7%) commonly occur during the procedure [3]. Adequate mucosal elevation by injecting submucosal injection agents is essential to remove flat or sessile type lesions to maintain a clear cutting view and reduce perforation [4]. The ideal submucosal injection agents in ESD should provide long-lasting mucosal cushion, convenient injectability, biocompatibility and biodegradability [5].

Normal saline (NS) is widely used in ESD procedures to create the submucosal fluid cushion (SFC) [6]. However, NS quickly diffuses from the injected site, requiring multiple injections for sufficient submucosal lifting and therefore prolonging the procedure time. Many other types of materials for SFC have been developed to overcome the defects of NS. Hyaluronic acid (HA), a non-sulfated glycosaminoglycan present in connective tissue, is used frequently for submucosal injection as a 0.4% solution [4,7]. The solution has a relatively high viscosity to provide long-lasting fluid cushion compared to other agents such as glycerol, dextrose water, and hydroxypropyl methylcellulose [8]. A HA-based product, the Blue Eye™ submucosal injection agent by The Standard Co., Ltd. in Republic of Korea, is CE approved. However, generally, if a solution has a high viscosity, it requires high injection force by endoscopists to inject the solution, complicating the procedure and reducing the success rate [9]. Recently, another submucosal injection agent was commercially developed by Boston Scientific, the composition of which is unknown [10]. However, this high viscosity gel is equipped with an especially designed delivery system for injection.

Recently, injectable hydrogels have been investigated as an SFC materials to reduce the injection force. For example, a photo-crosslinked chitosan hydrogel was studied to create an SFC in esophageal ESD [11]. A chitosan hydrogel was formed in situ by injecting a low viscous chitosan solution into the submucosa and irradiating with ultraviolet (UV) light for 5 min through an endoscopic accessory channel. However, this process requires additional time for polymerization and complicates the endoscopic procedure. Additionally, the partial chemical reaction by UV light causes an inhomogeneous hydrogel that results in inconvenience due to the procedure.

Thermosensitive injectable hydrogels have also been studied for SFC in ESD. Thermosensitive PLGA-PEG-PLGA triblock copolymers were devised for an injectable hydrogel [5,12]. The copolymers showed a drastic increase in viscosity at 30–35 °C suggesting that they may be an ideal substance to create an SFC. However, the copolymers have at least two times higher injection force at room temperature than glycerol fructose frequently used in the procedure. Moreover, it is difficult to synthesize the triblock copolymer and residual unreacted monomers in the synthesis may induce an inflammation to the tissue near the injection.

Poly(*N*-isopropylacrylamide) (PNIPAm) is a popularly used thermosensitive polymer due to its biocompatibility and a sharp phase transition at 32 °C [13,14]. However, the hydrogel should be removed surgically due to nonbiodegradability [15].

Pluronics, known as poloxamers, are thermosensitive synthetic block copolymers of hydrophilic poly(ethylene oxide) (PEO) and hydrophobic poly(propylene oxide) (PPO) with many advantages, such as biocompatibility and protein stability [14,16]. However, the fast degradation rate of Pluronics limits its biomedical applications, and it is frequently crosslinked with other materials to decelerate the degradation rate [17,18].

Alternatively, chitosan, an anti-bacterial, biocompatible, and biodegradable polymer, has been used extensively as a thermosensitive hydrogel for drug delivery systems [19,20,21,22,23]. Various types of chitosan thermosensitive hydrogels have been developed based on the gelling agent, such as NaHCO_3_ [21], K_2_HPO_4_ [20], and *β*-glycerol phosphate disodium salt (*β*-GP) [1], to form a viscous gel in situ at body temperature. Among these agents, *β*-GP naturally found in the body, is approved by the United States FDA for venous administration, and is widely used to form a chitosan thermosensitive hydrogel for its biocompatibility and ability induce a sol-to-gel transition at physiological pH and temperature [24,25,26,27,28]. However, to the best of our knowledge, the efficacy and feasibility of a chitosan and *β*-GP thermosensitive hydrogel has not been examined as a submucosal injection agent in ESD compared to other commercially available submucosal injection agent products. In the previous study, a chitosan-based aqueous solution was developed as a submucosal injection agent without gelling agent [29].

**Figure 1:**
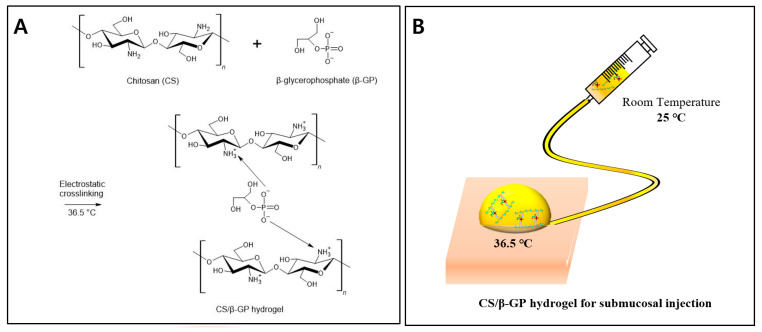
The schematic illustration of this study. (**A**) Chemical scheme of the reaction. Injectable thermosensitive chitosan (CS) solution with *β*-glycerophosphate (*β*-GP) produces CS/*β*-GP hydrogel at 36.5 °C. (**B**) Injectable thermosensitive chitosan solution at room temperature turns into a viscous hydrogel in the submucosa of the body resulting in submucosal fluid cushion (SFC) for endoscopic submucosal dissection (ESD).

In the present study, a chitosan thermosensitive hydrogel with *β*-GP was adopted as a submucosal injection agent (Figure 1) and the optimal concentration of *β*-GP was investigated for sufficient SFC in ESD comparable to commercially available products. The rheological characteristics, injectability, cytotoxicity, and preclinical application of the CS/*β*-GP hydrogel and other products were studied.

## 2. Materials and Methods

### 2.1. Preparation of the Thermosensitive Chitosan Solution

A chitosan solution was fabricated by dissolving 1% *w*/*v* chitosan (92.6% degree of deacetylation, provided by HEPPE MEDICAL Chitosan GmbH, Halle, Germany) in a 1% *w*/*v* lactic acid solution (Sigma Aldrich Korea, Yongin, Korea). A solution of 56% *w*/*v β*-glycerophosphate disodium salt hydrate (*β*-GP, Sigma Aldrich Korea) dissolved in distilled water was added and stirred with a magnetic stirrer to obtain a homogeneous and clear liquid solution [1]. The *β*-GP solution concentrations were 0–32% *w*/*v* (with 4% *w*/*v* interval).

### 2.2. Thermo-Sensitivity Evaluation

The thermo-sensitivity was examined by immersing solutions into 36–37 °C water to simulate body temperature. A thermometer data logger (CENTER 309, Center Technology Corp., New Taipei City, Taiwan) was used to measure the water temperature and boiled water was added to maintain the temperature. The solutions were examined 5 min after immersion and the vial was turned upside down to evaluate the gelation of the solution.

### 2.3. Rheological Evaluation

The rheological properties were assessed using a rotational rheometer (ARES-G2, TA instruments Ltd., New Castle, DE, USA) with a cone-plate (DIN-bob). For the temperature and time sweep tests, the storage modulus (G′), viscous modulus (G″) and viscosity (cP) were determined from the oscillating measurements at a 1-Hz frequency and a strain of 300%. The temperature was varied at a rate of 1 °C per minute for the temperature sweep test and the temperature was maintained at 36.5 °C for the time sweep test. The gelation temperature corresponds to the intersection of the G′ and G″ curves.

### 2.4. Injectability Evaluation

The injectability was evaluated with a universal testing machine (Withlab Co., Ltd., WL2100, Gunpo, Korea). Chitosan thermosensitive solutions and existing commercial submucosal injection agents, normal saline, Eleview^®^ (Cosmo Technologies Ltd., Pittsburgh, PA, USA), Blue Eye™ (The Standard Co., Ltd., Gunpo, Korea) and ORISE™ gel (Boston Scientific Ltd., Marlborough, MA, USA), were compared. A 5 mL luer-lock syringe (POONGLIM Pharmatech Inc., Gunsan, Korea) was filled and the syringe was connected with a 23G endoscopic needle (Olympus Co., Ltd., Tokyo, Japan) to measure the injection pressure in an environment similar to an endoscopic procedure (25 ± 2 °C, 45 ± 5%RH). The test speed was 100 mm/min and the load cell was 200 N. The test ended when the test solution was removed from the syringe. The average injection force during the test and maximum injection force were recorded for each solution.

### 2.5. Cytotoxicity

The cytotoxicity of the solutions was evaluated using water-soluble tetrazolium salt (WST-1, EZ-CYTOX, Daeillab Inc., Seoul, Korea). L929, a mouse fibroblast cell line, was cultured in Eagle’s minimum essential medium (α-MEM, Welgene, Gyeongsan, Korea) supplemented with 1% antibiotic-antimycotic solution (AA, Welgene) and 10% fetal bovine serum (FBS, Welgene). The cells were incubated in a humid incubator at 37 °C with 5% CO_2_. Log phase L929 cultures were harvested and seeded at a 2000 cells/well density in a 96 well plate. The cell culture plates were incubated for 24 h to achieve approximately 60–70% confluence and each well was then treated with the chitosan solution. After 24 h, the samples were treated with the WST-1 solution for 1 h. The optical density (OD) of the samples was measured using a microplate reader, followed by calculation of the cytotoxicity using the equation below.

$$\mathrm{Cytotoxicity}=\frac{{\mathrm{OD}}_{450\;\mathrm{nm}}\;\mathrm{sample}-{\mathrm{OD}}_{450\;\mathrm{nm}}\;\mathrm{blank}}{{\mathrm{OD}}_{450\;\mathrm{nm}}\;\mathrm{Control}-{\mathrm{OD}}_{450\;\mathrm{nm}}\;\mathrm{blank}}$$

### 2.6. Preclinical Evaluation

#### 2.6.1. Animal Model

Six, 40 ± 5 kg female pigs were used. Pigs were fasted from the evening before the experiment. On the day of the experiment, an intramuscular injection of azaperone (2–8 mg/kg), alfaxalone (2–6 mg/kg), Xylazine (1–3 mg/kg), Atropine (0.5 mg/kg) were administered for anesthesia induction. For intubation, alfaxalone (1–2 mg/kg), xylazine (0.5 mg/kg) were injected. Then, a 6.5 Fr sized endotracheal tube was used for intubation and maintained by Isoflurane 2.0%. After anesthesia, the internal body temperature of the pig was measured using an esophageal or rectal body cavity temperature probe (YSI 401, Advanced Industrial Systems, Inc., Prospect, KY, USA) by inserting into the stomach. The pigs were sacrificed immediately after the experiment. All animal experiments were approved by IACUC (KOREA-2020-0093) and conducted accordingly.

#### 2.6.2. Experiment Procedure

Two endoscopists participated in the experiment. An esophagogastroduodenoscope (GIF-Q260, Olympus Co., Ltd., Tokyo, Japan) was inserted into the stomach of the pig. The remaining residue was suctioned and the stomach was washed with normal saline so that the mucous membrane was clearly visible. One experimenter injected 5 mL of each solution into the antrum and body part of the submucosa using a 23 G endoscopic injector (NM-600L-0423, Olympus Co., Ltd.). The other experimenter observed the height and shape of the SFC created by the submucosal injection fluid and compared the change after 30 min.

### 2.7. Scanning Electron Microscope (SEM) Specimen

The surface morphology of submucosa after submucosal injection was observed using a field emission scanning electron microscope (FE-SEM) S-4700 (Hitachi, Tokyo, Japan). After injecting the agent into the submucosa layer, the mucosa and lamina propria layers were removed, and then, the remaining submucosa into small pieces of 1 cm or less was cut and fixed with a fixative (glutaraldehyde 2.5%, phosphate buffer 0.1 M, pH 7.24) for 2 h. Then, the specimen was dehydrated in alcohol at 60%, 70%, 80%, 90%, and 95% for 20 min each, and 100% for 30 min. After the drying process, the specimen was placed on the stub. After sputtering platinum, the tissue was observed using FE-SEM.

### 2.8. Statistical Analysis

Experimental results were presented as a form of mean ± standard deviation (SD). Analysis of variance (ANOVA, one-way) was employed to determine the significance of the differences in means. Fisher’s least significant difference test (LSD, *p* < 0.05) was used to compare the means of variables. LSD tests were performed using RStudio Version 1.4.1106 free software.

## 3. Results

### 3.1. Thermo-Sensitivity of Solutions

The thermo-sensitivity of the solutions was investigated using a water immersion test conducted at body temperature (Figure S1). The solutions were liquid state at room temperature regardless of the concentration of *β*-GP (Figure S1a). After water immersion, the liquid-to-gel transformation occurred within 10 min on higher *β*-GP concentration samples (Figure S1b). As the *β*-GP concentration increased, the extent of the gelation increased. The gelation did not occur below 16% of *β*-GP concentration at body temperature water and the gelation samples were reversed from gel to liquid within 48 h at room temperature (Table S1).

### 3.2. Rheological Evaluation

#### 3.2.1. Temperature Sweep

**Figure 2:**
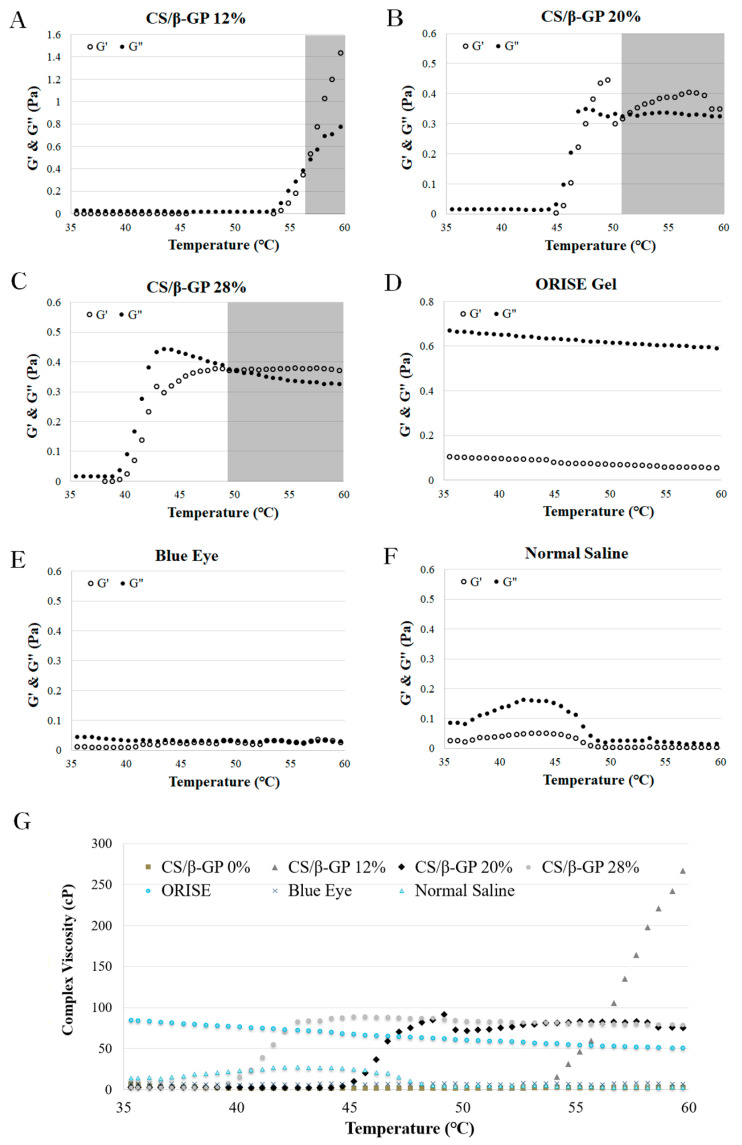
Temperature dependence of the loss modulus (G″, ●) and storage modulus (G′, ○) of (**A**) CS/*β*-GP 12%, (**B**) CS/*β*-GP 20% (**C**) CS/*β*-GP 28%, (**D**) ORISE™ gel, (**E**) Blue Eye™, and (**F**) normal saline. (**G**) Temperature dependence of the complex viscosity of all samples including CS/*β*-GP 0% as a control solution.

The first rheological results present the temperature sweep tests of CS/*β*-GP solutions at different compositions of *β*-GP (Figure 2A–C) and commercial submucosa injection agents (Figure 2D–F). In the CS/*β*-GP solutions, three *β*-GP concentrations, 12% *w*/*v* (A), 20% *w*/*v* (B), 28% *w*/*v* (C), were selected and evaluated for elastic modulus (G′), loss modulus (G″) and complex viscosity (Figure 2G) measurements from 35 to 60 °C. Overall, the CS/*β*-GP solutions present increasing behavior with different gelation temperatures while other commercial products remained constant or decreased. The gelation temperature of the CS/*β*-GP solutions were obtained by rheological results (56 °C, 51 °C and 49 °C, respectively, for CS/*β*-GP 12%, CS/*β*-GP 20% and CS/*β*-GP 28%).

In the CS/*β*-GP 12% solution, the values of G′ and G″ increased at 54 °C (Figure 2A). At the same temperature, the complex viscosity of the solution also increased (Figure 2G). The G′ value was greater than that of G″ before 56 °C; however, the G′ value becomes larger after 56 °C, and this result indicates that the gelation process occurred from liquid to solid at 56 °C. In the CS/*β*-GP 20% solution, the values of G′ and G″ increased at 45 °C (Figure 2B). At the same temperature, the complex viscosity of the solution also increased. (Figure 2G) In the modulus result, the intersection of G′ and G″ occurs two times near 47 °C and 51 °C. However, according to the trend of the increasing G′ value, the gelation temperature was 51 °C. In the CS/*β*-GP 28% solution, the values of G′ and G″ increased at 40 °C (Figure 2C). At the same temperature, the complex viscosity of the solution also increased (Figure 2G). In this sample, the gelation temperature was 49 °C at the intersection of G′ and G″. In the ORISE™ Gel, Blue Eye™ and normal saline conditions, the values of G′ and G″ remained constant or decreased except for a slight change in normal saline (Figure 2D–F). The complex viscosity of the solutions also remained constant or slightly changed (Figure 2G). Therefore, gelation did not occur in these samples because there was no intersection of G′ and G″.

#### 3.2.2. Time Sweep

**Figure 3:**
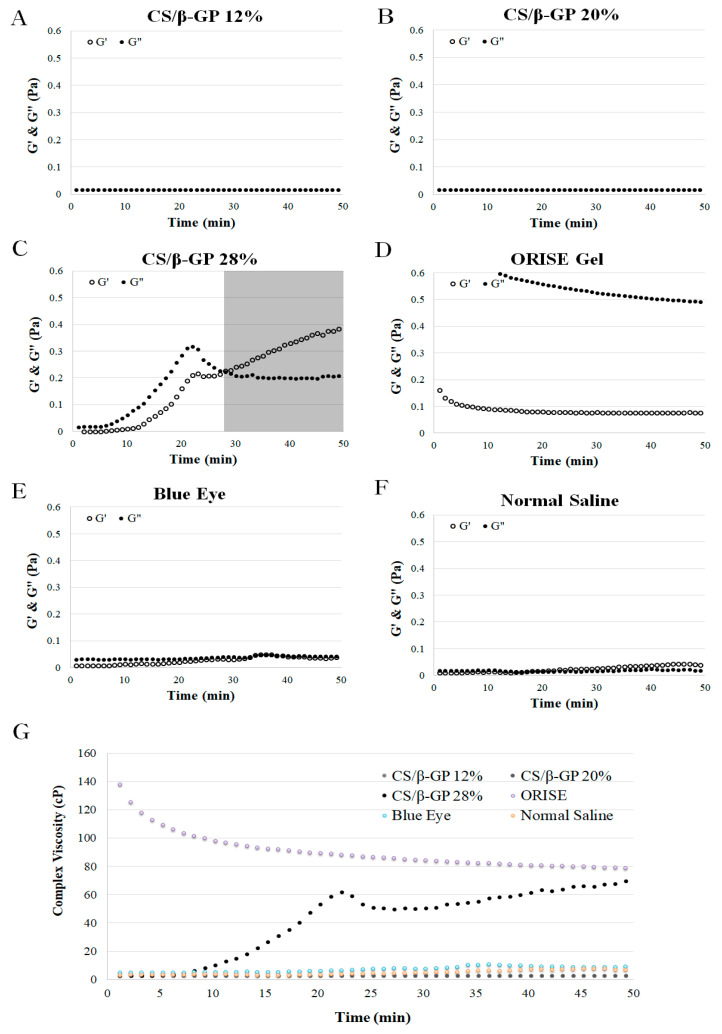
Time dependence of the loss modulus (G″, ●) and storage modulus (G′, ○) of (**A**) CS/*β*-GP 12%, (**B**) CS/*β*-GP 20%, (**C**) CS/*β*-GP 28%, (**D**) ORISE™ Gel, (**E**) Blue Eye™, an (**F**) normal saline conditions at 36.5 °C. (**G**) Time dependence of the complex viscosity of all samples.

The second rheological results present the time sweep tests of the CS/*β*-GP solutions at different compositions of *β*-GP (Figure 3A–C) and commercial submucosal injection agents (Figure 3D–F). Three *β*-GP concentrations, 12% *w*/*v* (A), 20% *w*/*v* (B), 28% *w*/*v* (C), were selected and evaluated for elastic modulus (G′), loss modulus (G″) and complex viscosity (Figure 3G) at 36.5 °C for 50 min. Only the *β*-GP 28% solution presented increasing G′ and G″ values while the other groups remained constant or decreased. The gelation time of the *β*-GP 28% sample was 28 min.

In the CS/*β*-GP 12% and 20% solutions, and other commercial submucosal injection agents, the G′, G″ values remained constant or decreased without intersection and the complex viscosity of the solutions at 36.5 °C remained constant or decreased with time (Figure 3A,B,D–G). Alternatively, in the CS/*β*-GP 28% solution, the G′ and G″ values increased over time at 36.5 °C. In the initial stage, the G″ value was greater than G′ indicating the solution was in a liquid state; however, after 28 min, the G′ value was greater, indicating the solution was gelled in a solid state (Figure 2C). The complex viscosity increased after 7 min, illustrating the viscosity was more than 60 times the initial value 23 min after the test (Figure 3G).

### 3.3. Injectability Evaluation

**Figure 4:**
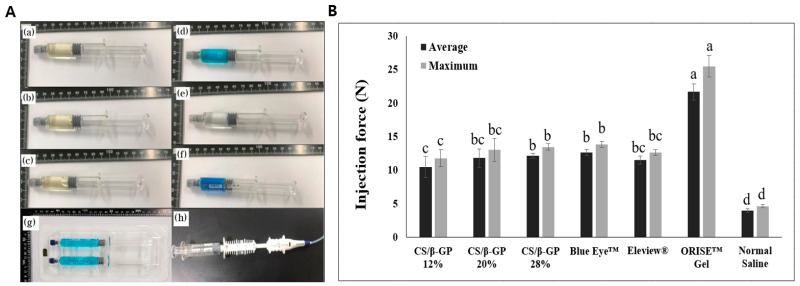
(**A**) Various submucosal injection agents to evaluate injection force; (**a**) CS/*β*-GP 12% (**b**) CS/*β*-GP 20% (**c**) CS/*β*-GP 28% (**d**) Eleview^®^ (**e**) normal saline (**f**) Blue Eye™ (**g**) ORISE™ gel (**h**) Endoscopic needle. (**B**) Injectability of the chitosan thermosensitive solutions (CS/*β*-GP 12%, CS/*β*-GP 20% and CS/*β*-GP 28%) and existing commercial submucosal injection agents. The average injection force and the maximum injection force were recorded for each sample (n = 5, ANOVA, Fisher’s LSD test, *p* < 0.05). Same letters indicate that there is no statistically significant difference between the samples.

An injectability test, reported in Figure 4, was performed to compare the injection pressure of chitosan thermosensitive solutions (CS/*β*-GP 12%, CS/*β*-GP 20% and CS/*β*-GP 28%) and other commercial submucosal injection agents in an environment similar to an endoscopic procedure.

The results indicated that both the average and maximum injection force increased as the *β*-GP concentration increased in the chitosan thermosensitive groups. The maximum injection pressures of each chitosan thermosensitive solution are 11.76 N, 13.03 N and 13.46 N (CS/*β*-GP 12%, CS/*β*-GP 20% and CS/*β*-GP 28%, respectively). The maximum injection pressure of the CS/*β*-GP 12% solution was less than all other commercial submucosal injection agent products, except normal saline (Blue Eye™ (13.81 N), Eleview^®^ (12.61 N) and ORISE™ gel (25.49 N). The maximum injection pressure of the CS/*β*-GP 20% and CS/*β*-GP 28% solutions was greater than that of Eleview^®^ but less than Blue Eye™ and ORISE™ gel. normal saline had the lowest injection pressure at less than 5 N.

### 3.4. Biocompatibility Evaluation

**Figure 5:**
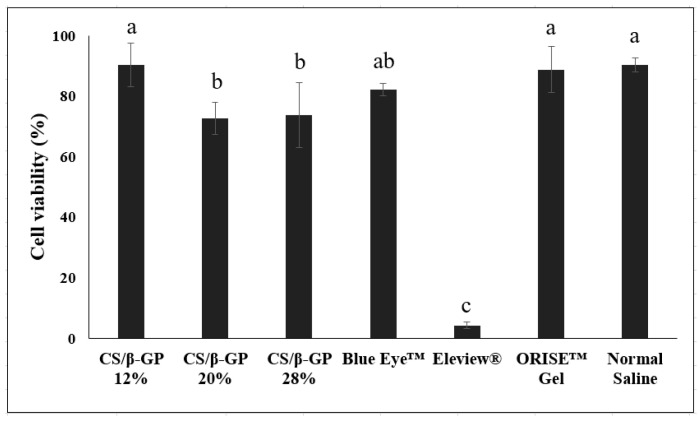
Cytotoxicity of the chitosan thermosensitive solutions (CS/*β*-GP 12%, CS/*β*-GP 20% and CS/*β*-GP 28%) and commercial submucosal injection agents (n = 4, ANOVA, Fisher’s LSD test, *p* < 0.05). Same letters indicate that there is no statistically significant difference between the samples.

An in vitro cytotoxicity test, reported in Figure 5, was performed to evaluate the biocompatibility of the chitosan thermosensitive solution. The CS/*β*-GP 12% solution showed 90.3% cell viability. As increasing the *β*-GP concentration to 20, 28%, the viability dropped to 73%. Among the commercial submucosal injection agents, normal saline showed the highest cell viability of 90.4%, followed by ORISE™ Gel and HA-based Blue Eye™. Chitosan thermosensitive solutions with a *β*-GP concentration of 12% had similar or greater cell viability than all of the other commercial products, except Eleview^®^, which showed the lowest cell viability at less than 5%.

### 3.5. Preclinical Evaluation

**Figure 6:**
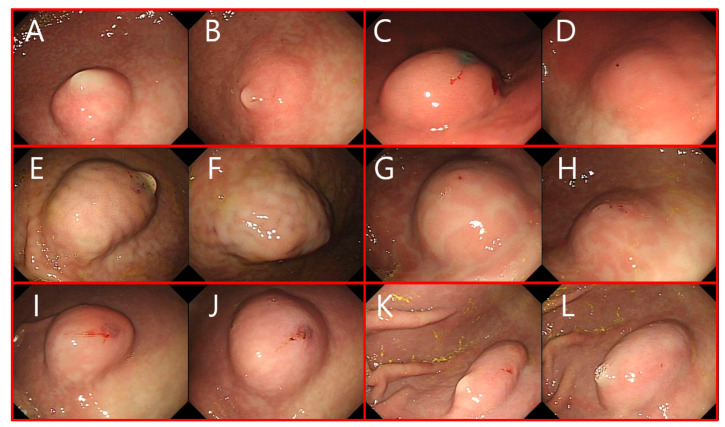
The SFC maintained by several submucosal injection agents right after injection (left side in each group; (**A**,**C**,**E**,**G**,**I**,**K**)) and 30 min after injection (right side in each group; (**B**,**D**,**F**,**H**,**J**,**L**)): (**A**,**B**) normal saline, (**C**,**D**) Eleview, (**E**,**F**) Blue Eye, (**G**,**H**) CS/*β*-GP 12%, (**I**,**J**) CS/*β*-GP 20%, and (**K**,**L**) CS/*β*-GP 28%.

The average temperature of the internal stomach was 37.2 ± 0.4 °C. The SFC made by injecting the solution did not show much difference in the shape or morphology. Most of the resulting SFCs had hemispherical shapes and the initial heights was similar. After 30 min, the height and shape of the SFCs from each solution were compared. Figure 6 shows the SFC of each solution at 0 and 30 min confirmed by an EGD. After 30 min, the height of the SFC made by normal saline nearly spread to its periphery and only slightly decreased. In addition, the Eleview^®^ sample maintained approximately half of its initial height. Alternatively, in the case of Blue Eye™, the height barely subsided, and the CS/*β*-GP 20% and CS/*β*-GP 28% solutions maintained similarly constant height. CS/*β*-GP 12% had a height reduction of about half, which was comparable to the Eleview^®^ height. Regarding the ease of injection, it was most difficult to inject Blue Eye™, followed by Eleview^®^. With the exception of normal saline, the chitosan thermosensitive solutions were the easiest to inject.

### 3.6. SEM Surface Examination

**Figure 7:**
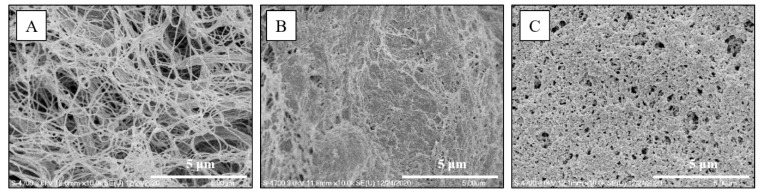
Submucosa surface examination using FE-SEM (**A**) normal saline, (**B**) Blue Eye, (**C**) CS/*β*-GP 20%. (Scale bar is 5 μm).

The surface morphologies of submucosa after various submucosal injections; normal saline, Blue Eye, and CS/*β*-GP 20%, reported in Figure 7, were observed using FE-SEM. In the case of the surface of submucosa subjected to ESD with normal saline, collagen fiber bundles intertwined into a network, and many empty spaces were observed between the collagen fibers resulting in a porous structure (Figure 7A). In the case of the Blue Eye™, interconnectivity between collagen fibers was higher than that of normal saline, but the porosity was reduced due to high interconnectivity (Figure 7B). In the case of the CS/B-GP 20% hydrogel, the interconnectivity between collagen fibers was high and the porosity was also maintained (Figure 7C).

## 4. Discussion

Side effects of ESD, such as bleeding and perforation, restrict its application [30,31]. SFCs are essential to minimizing the side effects of ESD; however, the widely used normal saline has inadequate cushioning maintenance. Other commercial products have higher viscosity than normal saline, which provides a longer lasting SFC, but also requires a high injection pressure that can be difficult to use. Therefore, thermosensitive hydrogels are a promising option for submucosal injection agents as a low viscous liquid state during injection that becomes a semi-solid, gel-like state after injecting into the submucosa [5].

Chitosan-based thermosensitive hydrogels are extensively used for their biocompatible, biodegradable, anti-bacterial and hemostatic properties [1,32]. Several studies have indicated that a *β*-GP derived chitosan thermosensitive hydrogel is promising as a biomaterial for medical applications [33,34]. However, no studies have been performed to find the proper concentration of *β*-GP for its application in ESD. In the present study, a chitosan thermosensitive solution with *β*-GP was adopted as a submucosal injection agent and the concentration of *β*-GP was investigated to determine the optimal condition for sufficient SFC in ESD compared to commercially available products. The rheological characteristics, injectability, cytotoxicity and preclinical application of the CS/*β*-GP solutions and other products were studied. The fabricated CS/*β*-GP hydrogels with *β*-GP concentrations of 0–32% *w*/*v* were a low viscosity solution at room temperature and some hydrogels with high *β*-GP concentration became opaque at body temperature, ~37 °C (Figure S1). The gelation was stronger as the concentration of *β*-GP increased. Hydrogen bonding of polyols such as *β*-GP reduce as the temperature increases, which results in weak hydration of the chitosan chain, leading to gelation [35]. High concentrations of *β*-GP neutralize the chitosan chain more than low concentrations, leading to more hydrophobic interactions and gelation when the temperature increases.

Three samples (*β*-GP concentrations of 12%, 20%, and 28%) were selected for the characterization and feasibility testing. Samples with *β*-GP concentrations greater than 30% were excluded due to gelation at room temperature and samples with less than 10% *β*-GP were excluded due to insufficient gelation after immersion in body temperature water (Figure S1). Fabricated CS/*β*-GP 12, 20, 28% solutions were stable for 48 h on the room temperature (Figure S2). Rheological characteristics of the thermosensitive chitosan and commercial products were investigated with a temperature sweep of 30–65 °C and time sweep at 36.5 °C for 50 min (Figure 3 and Figure 5). Only the chitosan thermosensitive solutions showed reverse phase transitions with increasing viscosity over temperature, while the viscosity of commercial products, such as normal saline, HA-based Blue Eye™ and ORISE™ gel, slightly changed (Figure 2). The gelation temperature is derived from the temperature at which elastic modulus (G′) and loss modulus (G″) meet, but the temperature at which the viscosity of the hydrogel increases is 5 to 10 °C lower than the gelation temperature [35].

If the solution has a high viscosity in vivo, then the duration of the SFC is enhanced but also has a high injection pressure [36,37]. Therefore, an ideal submucosal injection agent is a low viscous state before endoscopic injection, which is then turned into a gel-like state with high viscosity when injected into the submucosa. In the CS/*β*-GP 28% sample that viscosity increased at body temperature, 37 °C (Figure 2C,G). Therefore, only the CS/*β*-GP 28% solution showed a reverse phase transition in the time sweep test at 36.5 °C while other CS/*β*-GP solutions and commercial products remained or decreased in viscosity (Figure 3). Although the viscosity of CS/*β*-GP 28% was less than that of the ORISE™ gel, the injection pressure of the ORISE™ gel was two times greater than that of CS/*β*-GP 28% (Figure 4). The ORISE™ gel was omitted due to its high injection force in the preclinical evaluation.

The cytotoxicity of the chitosan thermosensitive solutions and other commercial products showed the presumed in vivo biocompatibility when injected into the submucosa (Figure 5). CS/*β*-GP 12% solution had a highest cell viability similar to other commercial products, except Eleview^®^, which had the lowest cell viability presumed to be caused by emulsifier or oil components. Though CS/*β*-GP 20%, 28% showed slightly lower cell viability than other commercial products, CS/*β*-GP solutions have sufficient biocompatibility in vivo (>70% cell viability) [38].

When observed with an endoscope in the preclinical study, the SFC of the antrum tended to sink faster than the stomach body. After 30 min, the SFC height of the antrum was always less than that of the body or all solutions. Given the same amount of submucosal fluid was injected, the submucosa layer of the antrum is thicker than the body and spreads faster [24]. Chitosan thermosensitive solutions showed that the SFC height in the body was more constant after 30 min with increasing *β*-GP content (Figure 6). In addition, SFC made by CS/*β*-GP 20% and 28% were similar to that of HA-based Blue Eye™. HA is an outstanding FDA-approved submucosal injection solution, though expensive. However, when the *β*-GP content in the chitosan was greater than 20%, the SFC hardly subsided for at least 30 min and the effect was maintained. Given that the internal body temperature does not change over time, the developed temperature-sensitive chitosan is an effective and efficient hydrogel for ESD. This property could facilitate endoscopic procedures by reducing multiple injections for SFC during operation and reducing the procedure time. This result correlated with the rheological and injectability results.

Moreover, the surface morphology of submucosa with CS/*β*-GP 20% showed high interconnectivity between collagen fibers and adequate porosity (Figure 7C). This property could facilitate cell adhesion, migration, and subsequent cellular events after the ESD procedure [39].

The limitation of the study was that ESD procedures with the chitosan thermosensitive solutions were not performed in the preclinical study. Further study is required to reveal the clinical safety and effectiveness in ESD procedures compared to common submucosal injection agents.

## 5. Conclusions

Chitosan thermosensitive solutions were developed as a submucosal fluid cushion to find the optimal *β*-GP concentration in ESD compared with commercial submucosal injection products. CS/*β*-GP 28% was the best thermosensitive solution as it had a low viscous state at room temperature and showed high viscosity with reverse phase transition in body temperature in the rheological results while other products showed no thermosensitivity. Cytotoxicity data showed CS/*β*-GP 12%, 20%, and 28% were biocompatible. In the preclinical study, CS/*β*-GP 20%, 28% showed high maintenance of submucosal fluid cushion similar to hyaluronic acid base Blue Eye™. Therefore, developed chitosan thermosensitive solution is suitable as an ideal submucosal injection agent.

## Data Availability

All the experimental data herein presented are available on request from the corresponding author.

## Supplementary Materials

The following are available online at https://www.mdpi.com/article/10.3390/polym13111696/s1, Figure S1: Chitosan thermosensitive solutions on room temperature before water immersion and after 10 min of immersion in body temperature water., Table S1: Gel formation time at body temperature water and time from gel to liquid at room temperature of the chitosan hydrogels according to various *β*-GP concentrations., Figure S2: Stability of the solutions on room temperature for 48 hr.
