# Supplementary material for: Injectable Thermosensitive Chitosan Solution with β-Glycerophosphate as an Optimal Submucosal Fluid Cushion for Endoscopic Submucosal Dissection
Source: Polymers (Basel). 2021 May 22;13(11):1696. doi: 10.3390/polym13111696 (PMC8196954; doi:10.3390/polym13111696)
Supplement: Supplementary file 1 [file polymers-13-01696-s001.zip › polymers-1217191-supplementary materials.pdf]

polymers

MDPI

Article

Injectable Thermosensitive Chitosan Solution with β-Glycerophosphate as an Optimal Submucosal Fluid Cushion for Endoscopic Submucosal Dissection

Seung Jeong [1,†], Han Jo Jeon [2,†], Kyoung-Je Jang [3,4], Sangbae Park [1], Hyuk Soon Choi [2,*] and Jong Hoon Chung [1,5,*]

[1] Department of Biosystems & Biomaterials Science and Engineering, Seoul National University, Seoul 08826, Korea; jsw3055@snu.ac.kr (S.J.); sb92park@snu.ac.kr (S.P.)
[2] Division of Gastroenterology and Hepatology, Department of Internal Medicine, Korea University College of Medicine, Seoul 02841, Korea; roadstar82@naver.com
[3] Division of Agro-system Engineering, College of Agriculture and Life Science, Gyeongsang National University, Jinju 52828, Korea; kj_jang@gnu.ac.kr
[4] Institute of Agriculture & Life Science, Gyeongsang National University, Jinju 52828, Korea
[5] Research Institute for Agriculture and Life Sciences, Seoul National University, Seoul 08826, Korea
* Correspondence: author: mdkorea@gmail.com (H.S.C.); jchung@snu.ac.kr (J.H.C.); Tel: +82-2-920-6555 (H.S.C.); +82-2-880-4601 (J.H.C.); Fax: +82-2-953-1943 (H.S.C.); +82-2-873-2049 (J.H.C.)
† Both authors contributed equally to this work.

Supplementary Materials

Citation: Jeong, S.; Jeon, H. J.; Jang, K.-J.; Park, S.; Choi, H. S.; Chung, J. H. Injectable Thermosensitive Chitosan Solution with β-Glycerophosphate as an Optimal Submucosal Fluid Cushion for Endoscopic Submucosal Dissection. *Polymers* **2021**, *13*, 1696. https://doi.org/10.3390/polym13111696

Academic Editor: Luminita Marin

Received: 26 April 2021
Accepted: 19 May 2021
Published: 22 May 2021

Publisher's Note: MDPI stays neutral with regard to jurisdictional claims in published maps and institutional affiliations.

Copyright: © 2021 by the authors. Licensee MDPI, Basel, Switzerland. This article is an open access article distributed under the terms and conditions of the Creative Commons Attribution (CC BY) license (http://creativecommons.org/licenses/by/4.0/).

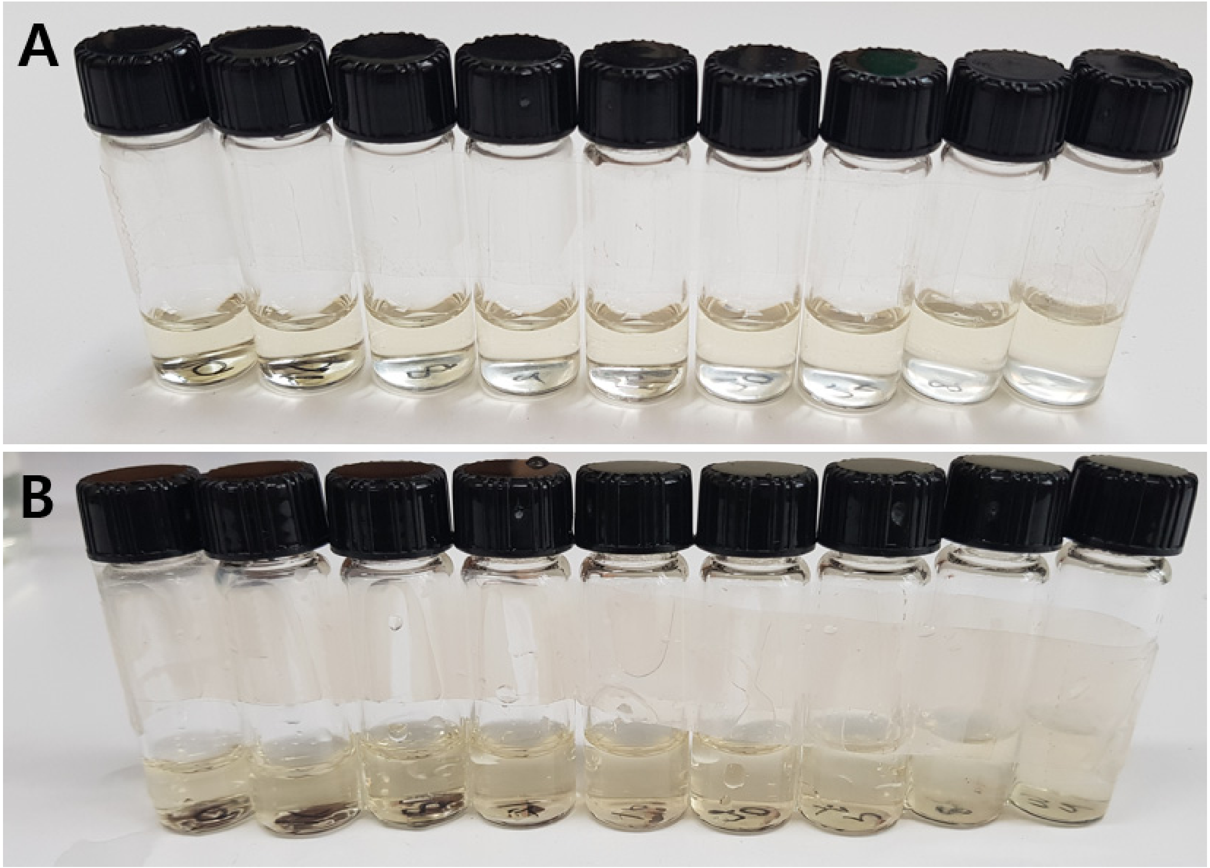

Figure S1. (A) Solutions on room temperature before water immersion. (B) Solutions after 10 minutes of immersion in body temperature water. As the β-GP concentration increases, the solutions became gradually opaque.

Table S1. Gel formation time at body temperature water and time from gel to liquid at room temperature of the chitosan hydrogels according to various β-GP concentrations.

	Gel formation time at body temperature water	Time from gel to liquid at room temperature
CS/β-GP 0 %	N.D.	N.D.
CS/β-GP 4 %	N.D.	N.D.
CS/β-GP 8 %	N.D.	N.D.
CS/β-GP 12 %	N.D.	N.D.
CS/β-GP 16 %	N.D.	N.D.
CS/β-GP 20 %	30 min	< 48 hr
CS/β-GP 24 %	15 min	< 48 hr
CS/β-GP 28 %	10 min	< 48 hr
CS/β-GP 32 %	8 min	< 48 hr

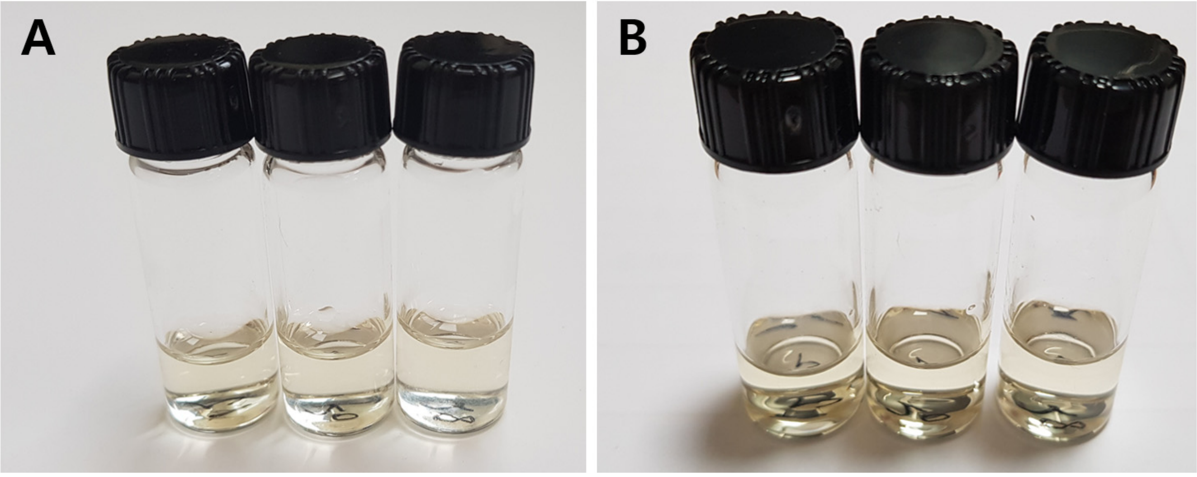

Figure S2. (A) CS/β-GP 12 %, 20%, 28% solutions on room temperature. (B) Solutions after 48 hr on room temperature. The solutions were stable at the room temperature for 48 hr.
